# *N*-3-Oxo-Octanoyl Homoserine Lactone Primes Plant Resistance Against Necrotrophic Pathogen *Pectobacterium carotovorum* by Coordinating Jasmonic Acid and Auxin-Signaling Pathways

**DOI:** 10.3389/fpls.2022.886268

**Published:** 2022-06-14

**Authors:** Fang Liu, Qian Zhao, Zhenhua Jia, Siyuan Zhang, Juan Wang, Shuishan Song, Yantao Jia

**Affiliations:** ^1^State Key Laboratory of Plant Genomics, Institute of Microbiology, Chinese Academy of Sciences, Beijing, China; ^2^College of Life Sciences, University of Chinese Academy of Sciences, Beijing, China; ^3^Biology Institute, Hebei Academy of Sciences, Shijiazhuang, China; ^4^Shijiazhuang Academy of Agricultural and Forestry Sciences, Shijiazhuang, China; ^5^Julu Institute of Applied Technology, Xingtai, China

**Keywords:** jasmonic acid, auxin, AHL, *Pectobacterium carotovorum*, priming

## Abstract

Many Gram-negative bacteria use small signal molecules, such as *N*-acyl-homoserine lactones (AHLs), to communicate with each other and coordinate their collective behaviors. Recently, increasing evidence has demonstrated that long-chained quorum-sensing signals play roles in priming defense responses in plants. Our previous work indicated that a short-chained signal, *N*-3-oxo-octanoyl homoserine lactone (3OC8-HSL), enhanced Arabidopsis resistance to the hemi-biotrophic bacteria *Pseudomonas syringae* pv. *tomato* DC3000 through priming the salicylic acid (SA) pathway. Here, we found that 3OC8-HSL could also prime resistance to the necrotrophic bacterium *Pectobacterium carotovorum* ssp. *carotovorum* (*Pcc*) through the jasmonic acid (JA) pathway, and is dependent on auxin responses, in both Chinese cabbage and Arabidopsis. The subsequent *Pcc* invasion triggered JA accumulation and increased the down-stream genes’ expressions of JA synthesis genes (*LOX*, *AOS*, and *AOC*) and JA response genes (*PDF1.2* and *VSP2*). The primed state was not observed in the Arabidopsis *coi1*-*1* and *jar1-1* mutants, which indicated that the primed resistance to *Pcc* was dependent on the JA pathway. The 3OC8-HSL was not transmitted from roots to leaves and it induced indoleacetic acid (IAA) accumulation and the *DR5* and *SAUR* auxin-responsive genes’ expressions in seedlings. When Arabidopsis and Chinese cabbage roots were pretreated with exogenous IAA (10 μM), the plants had activated the JA pathway and enhanced resistance to *Pcc*, which implied that the JA pathway was involved in AHL priming by coordinating with the auxin pathway. Our findings provide a new strategy for the prevention and control of soft rot in Chinese cabbage and provide theoretical support for the use of the quorum-sensing AHL signal molecule as a new elicitor.

## Introduction

Many bacteria use small signal molecules to communicate with each other and modulate their collective behavior, a process called quorum sensing (Taga et al., 2003; Reading and Vanessa, 2010). The most common quorum-sensing signal molecules in Gram-negative bacteria are *N*-acyl-homoserine lactones (AHLs; Sharma et al., 2020). AHLs molecules have varied acyl chain lengths (from 4 to 18 carbons) and substitutions of hydroxyl (OH) or oxo (O) groups at the chain’s γ position (Sharma et al., 2020). To date, over 30 types of AHLs have been identified from more than 70 species of Gram-negative bacteria.

Accumulating evidence indicates that bacterial AHLs are perceived by plant cells and modulate plant growth and development, as well as the responses to abiotic and biotic stresses, particularly those involved in plant immunity (Palmer et al., 2014). Mathesius et al. (2003) reported that the treatment of *Medicago truncatula* with two AHLs, *N*-3-oxo-dodecanoyl-homoserine lactone (3OC12-HSL) and *N*-3-oxo-hexadecanoyl-homoserine lactone (3OC16-HSL), resulted in the differential expression of proteins involved in the processes of flavonoid synthesis, hormone metabolism, and oxidative stress by two-dimensional gel electrophoresis (2D-PAGE). Our previous proteomic analysis showed that differentially expressed proteins were involved in carbon metabolism, protein biosynthesis, and plant resistance after plants were pretreated with *N*-3-oxo-octanoyl homoserine lactone (3OC8-HSL; Miao et al., 2012). The exposure of Arabidopsis roots to *N*-hexanoyl-homoserine lactone (C6-HSL), *N*-3-oxo-hexanoyl-homoserine lactone (3OC6-HSL), and 3OC8-HSL promotes primary root growth, whereas treatment with *N*-decanoyl-homoserine lactone (C10-HSL) inhibits primary root growth, but promotes lateral root and root hair formation in Arabidopsis (Ortíz-Castro et al., 2008; Rad et al., 2008; Liu et al., 2012; Schenk et al., 2012; Zhao et al., 2016; Shrestha et al., 2020). Inoculation with AHL-producing *Burkholderia graminis* M14 enhances the ability of tomatoes to tolerate salt stress. Similarly, 3OC6-HSL enhances salt tolerance in Arabidopsis and wheat (Zhao et al., 2020). Exposure to AHLs can elicit plant immunity. Several long-chain AHLs have been shown to induce AHL-priming for enhancing resistance against biotrophic and hemi-biotrophic pathogens in *Arabidopsis thaliana*, *Medicago truncatula*, and *Hordem vulgare* (Mathesius et al., 2003; Schikora et al., 2011; Schenk et al., 2012, 2014; Zarkani et al., 2013; Shrestha et al., 2020). The *N*-3-oxo-tetradecanoyl-homoserine lactone (3OC14-HSL)-mediated resistance priming in plants involves mitogen-activated protein kinase 6 (MPK6) activation, phenolic compound accumulation, lignin, and callose deposition (Schikora et al., 2011; Schenk et al., 2012, 2014). Some short-chain AHLs such as C6-HSL and *N*-3-hydroxybutyl-homoserine (C4-HSL) increase the expressions of salicylic acid (SA)- and ethylene-responsive defense genes and the SA accumulation in tomato plants (Schuhegger et al., 2006). Root inoculation with C4-HSL- and C6-HSL-producing *Serration plymuthica* protects plants from infection by *Botrytis cinerea* (Pang et al., 2009).

Priming is regulated by a complex network, which allows plants to activate defense responses in a faster and stronger manner as a consequence of triggering stimuli (Mauch-Mani et al., 2017). Many chemicals can induce priming, such as SA, benzothiadiazole (BTH), β-aminobutyric acid, pipecolic acid, jasmonic acid (JA), and volatile organic compounds (VOCs; Conrath et al., 2002; Martinez-Medina et al., 2016). The SA was the first synthetic compound shown to prime defense responses (Kauss et al., 1992) and effectively induce resistance against major fungal and bacterial pathogens in various crops (Kessmann et al., 1994). BTH acts as a priming agent in plant defense leading to a reduction in the penetration and development of the root-knot nematode *Meloidogyne incognita* in susceptible tomato roots (Veronico et al., 2018). β-Aminobutyric acid is a non-protein amino acid that primes the plants defense system to protect plants from various microbial pathogens (Thevenet et al., 2016). In Arabidopsis, the SA-dependent signaling pathway is considered to be effective mainly against biotrophic pathogens, such as the oomycete *Hyloperonospora*, the fungus *Erysiphe orontii*, and the hemi-biotrophic bacterium *Pseudomonas syringae*, and the JA-dependent defense response is considered to be effective mainly against necrotrophic microbial pathogens, such as the fungus *B. cinerea* and the bacterium *Pectobacterium carotovorum* ssp. *carotovorum* (*Pcc*; Norman-Setterblad et al., 2000; Zimmerli et al., 2000; Friml et al., 2003).

Using AHL-producing and AHL-negative strains, researchers have demonstrated the important role of C4-HSL and C6-HSL in the induction of resistance against necrotrophic pathogens in plants (Schuhegger et al., 2006; Pang et al., 2009). 3OC14-HSL enhances plant systemic resistance to biotrophic and hemi-biotrophic pathogens, such as *Golovinomyces orontii*, *Blumeria graminis* f. sp*.hordei*, and *P. syringae*, but not to necrobiotrophic pathogens, including *B. cinerea* and *Plectosphaerella cucumber* (Schikora et al., 2011). In contrast, Hu et al. (2018) found that C10-HSL treatment induced systemic immunity and protected tomatoes from infection by the necrotrophic fungus *B. cinere*a. His results showed that C10-HSL-induced resistance against *B. cinerea* was mainly dependent on the JA-signaling pathway. These contradictory results may indicate the complexity of the interaction outcome between plants and bacteria modulated by AHLs. We recently demonstrated the function of 3OC8-HSL in priming against hemi-biotrophic bacterial pathogen (Liu et al., 2020). However, whether 3OC8-HSL primes plant resistance to necrotrophic bacteria and the mechanism by which 3OC8-HSL induces resistance remain unknown.

The Gram-negative bacterium *Pcc* is a species of necrotrophic pathogen that causes soft-rot disease in a wide variety of plants (Perombelon and Kelman, 1980). In the present study, we found that the expression levels of genes involved in JA and auxin pathways were induced by 3OC8-HSL treatment. Pretreatment in which 10 μM 3OC8-HSL was added to the roots for 48 h decreased the disease symptoms and the *Pcc* growth on leaves of both Arabidopsis and Chinese cabbage. We investigated the roles of auxin in the interactions between *Arabidopsis thaliana* plants and their necrotrophic pathogen *Pcc* after pretreatment with AHL. Our results suggested that AHL contributed to the enhanced resistance in systemic leaves and provided evidence supporting the hypothesis that the JA pathway is involved in AHL priming by coordinating with the auxin pathway.

## Materials and Methods

### Plant Growth and Chemicals

*Arabidopsis thaliana* ecotype Columbia-0 (*Col-0*) was used throughout this study. The Arabidopsis mutants and transgenic lines used in the study are in the *Col-0* background. Seeds of the T-DNA insertion null mutants of *coronatine insensitive 1-1*, *coi1-1* (CS4144), and *jar1-1* (CS8072) were obtained from The Arabidopsis Information Resource (TAIR)^1^. The *jar1-1* mutant is compromised in the synthesis of jasmonic acid–isoleucine (JA-Ile), the active compound in JA signaling, whereas *coi1-1* is defective in JA perception (Chini et al., 2007). Some of the transgenic plant materials have been described previously, as follows: *DR5::GFP, DR5::GUS* (Sun et al., 2010), *PIN1::PIN1-GFP* (Benková et al., 2003) and *PIN3::PIN3-GFP* (Blilou et al., 2005). The Chinese cabbage used is the homozygous inbred line ‘*A03*’ that has light green leaves. The cabbage is normally grown and cultivated in the greenhouse. The experiment was carried out on potted cabbage approximately 20 days after germination. For pathogenicity, transcriptional, and biochemical analyses, the plants were cultivated using a hydroponic system. Arabidopsis seeds were surface sterilized with 75% (v/v) ethanol for 1 min and 30% (v/v) NaClO for 5 min. After washing five times with distilled water, seeds were germinated and grown on agar plates containing Murashig and Skoog medium (MS) at pH 5.8. Plants were placed in a growth chamber having a 16-h light:8-h dark photoperiod and 4,000-Lux light intensity at 22 ± 2°C. When the seedlings were grown to the two-leaf stage and roots reached 2 cm in length, the plants were transplanted into a plastic basin (a modified Eppendorf holder covered with parafilm:18 cm × 11 cm) containing 400 ml of Hoagland medium, which was exchanged every 2 days. AHLs were added directly into the medium.

### AHLs Pretreatment

The four shorter acyl chain AHLs (C6-HSL, 3OC6-HSL, 3OC8-HSL, and *N*-octanoyl-homoserine lactone C8-HSL) were dissolved independently in distilled water and the two longer acyl-chain AHLs C10-HSL and 3OC14-HSL, detected in this study, were dissolved in acetone. They were all purchased from Sigma-Aldrich (Taufkirchen, Germany) and stored in dry condition. They were diluted independently into 10 mM stock solutions in distilled water or acetone and adjusted to pH 5.0 just before use. All the compound solutions were sterilized by passing them through a 0.22 μm filter. AHLs were added directly into the hydroponic system. Plants were pretreated for 2 days. All the experiments were performed using the unpretreated plants.

### Microarray Analysis

Seventeen-day-old seedlings were cultured in Hoagland medium with or without 10 μM 3OC8-HSL. Plants were harvested at 24 h after the 3OC8-HSL pretreatment. Total RNA was extracted from pretreated and unpretreated plants using the RNAiso Plus reagent (TaKaRa, dalian, China) and purified using a NucleoSpin RNA clean-up kit (Macherey Nagel) in accordance with the manufacturers’ instructions. The probes were prepared using a CapitalBio cRNA-amplified labeling kit (Capitalbio Corp.) and fluorescently labeled with Cy5-dCTP and Cy3-dCTP (GE Healthcare). The 29k Arabidopsis Genome Arrays (Capitalbio Corp.) were prepared in accordance with the *A. thaliana* Genome Oligo Set (version 3.0; Operon). After hybridization, the arrays were scanned using a LuxScan 10KA two-channel laser scanner (CapitalBio Corp.) and analyzed using the LuxScan 3.0 software (CapitalBio Corp.). Each data point represents the average of three independent experiments. A two-fold increase (ratio > 2.0) or a two-fold decrease (ratio < 0.5) in the expression of pretreated plants compared with untreated plants was considered as a differential expression, corresponding to the upregulation or downregulation, respectively, in response to the AHL. The gene annotation and functional classification were performed using the Molecule Annotation System v3.0 and the Gene Ontology tool at TAIR. The microarray data discussed in the present study have been deposited in NCBI GEO and were released as GEO Series accession number GSE197485. The 3OC6-HSL Microarray data were published and are accessible through the GEO Series accession number GSE78079. Gene expression profiling and functional analyses were plotted using http://www.bioinformatics.com.cn, a free online platform for data analysis and visualization.

### Pathogenicity Tests

Arabidopsis plants were inoculated with the bacterial pathogen. *Pcc* was cultured overnight in Luria-Bertani medium (LB) until colony forming units (CFUs) reached 10^9^ CFU/ml. The cells were collected by centrifugation, washed in 10 mM MgCl_2_, and re-suspended in 10 mM MgCl_2_. The inoculation solution was adjusted to 10^7^ CFU/ml. Plants grown in the hydroponic system were spray-inoculated with a bacterial solution containing 0.02% Silwet L-77 uniformly. After 1, 6, 12, 24, 36, and 48 h, 100 mg leaf tissue was harvested and homogenized in 1 mM MgCl_2_. Samples were diluted and plated for CFU counting. Each of the six independent biological experiments was conducted with three technical replications. Chinese cabbage was inoculated with the bacterial pathogen. Briefly, petioles of the third leaves (from inside to outside) of 7- to 8-leaf plants were lightly scored (through the epidermis) with a sterile scalpel and inoculated with 5–10 μL of a uniform bacterial suspension made from cultures, which were labeled “*in vivo*”. Similarly, the third leaves were cut into 5.5-cm-diameter disks and placed in closed 9-cm-diamater petri dishes containing two layers of moist filter paper to maintain high humidity and then inoculated with 5–10 μL of bacterium suspension. They were then placed in an incubator (at 28°C with 90% relative humidity). These cultures were designated as “*in vitro*”. The inoculation concentration was 10^8^CFU/mL and the disease phenotype was investigated at 48 h after inoculation (Liu et al., 2019).

### qRT-PCR

The 3OC8-HSL-pretreated or unpretreated Arabidopsis seedlings were collected at 0, 6, 12, and 24 h post-inoculation with *Pcc*. The total RNA of homogenized plant tissues was extracted using the RNA plus reagent purchased from TaKaRa. Briefly, the cDNA was synthesized using the PrimeScript RT Reagent Kit with gDNA Eraser (TaKaRa) in accordance with the manufacturer’s instructions. For the relative quantification of gene expression, the comparative CT method (Livak and Schmittgen, 2001) with a 7,500 Real Time PCR System (Applied Biosystems, Foster City, CA, United States) was used. PCR amplification was performed in a total volume of 20 μL containing 5 μL diluted cDNA, 0.4 μL of each primer (10 μM), and 10 μL SYBR Premix Ex Taq™ (TaKaRa). The following qRT-PCR thermal cycling program was employed: 10 s at 95°C, 40 cycles of 5 s at 95°C, and 34 s at 60°C. The amount of target gene was normalized to the endogenous reference gene *Actin2/8*. Each data point represented the average of three independent experiments. For technical controls, each qRT-PCR experiment was repeated four times on the same 96-well plate. qRT-PCR was performed using the primers listed in Supplementary Table 1.

### Jasmonic Acid Measurement

Extraction and quantification of free JA were performed using 3OC8-HSL-pretreated Arabidopsis seedlings grown in the hydroponic system at 24 h after inoculation with *Pc*c. Plant tissues were frozen and ground in liquid N_2_. As an internal standard, 5 μL of 10 μg/mL dihydro-JA was added to the frozen tissue (0.5 g). In addition, 5 mL of 80% cold methanol was added, the samples were vortexed for 1 min for fully dissolving the powder, and extracted at 4°C overnight. The samples were centrifuged at 4,000 g for 15 min at 4°C. The supernatant was removed with a 1.5 mL syringe and passed through a 0.22 μM organic filter. The filtrate was prepared for HPLC-MS analysis. The control was 1 μg/mL JA standard. Chromatography was performed on a Waters1525 HPLC system (Waters Technologies). Chromatographic separation was achieved on an Inertsil ODS C18 column (50 mm × 4.6 mm, 5 μm, GL Sciences, Tokyo, Japan).

### Laser Scanning Confocal Microscopy

Propidium iodide was used to stain the plant cell wall. Examination of Green Fluorescent Protein (GFP) fluorescence intensity was performed using a laser scanning confocal microscope (excitation, 488 nm; emission, 500-550 nm; Leica SP8).

### Statistical Analysis

For all the experiments, the overall data were statistically analyzed using the DPS v7.05 program. ANOVA test was used to determine plant defense responses to 3OC8-HSL in different genotypes, including wild-type (*Col-0*), *coi1-1*, and *jar1-1*. All the data were represented as mean ± SD of three or six independent experiments.

## Results

### 3OC8-HSL Protects Arabidopsis From *Pcc* Infection

**FIGURE 1:**
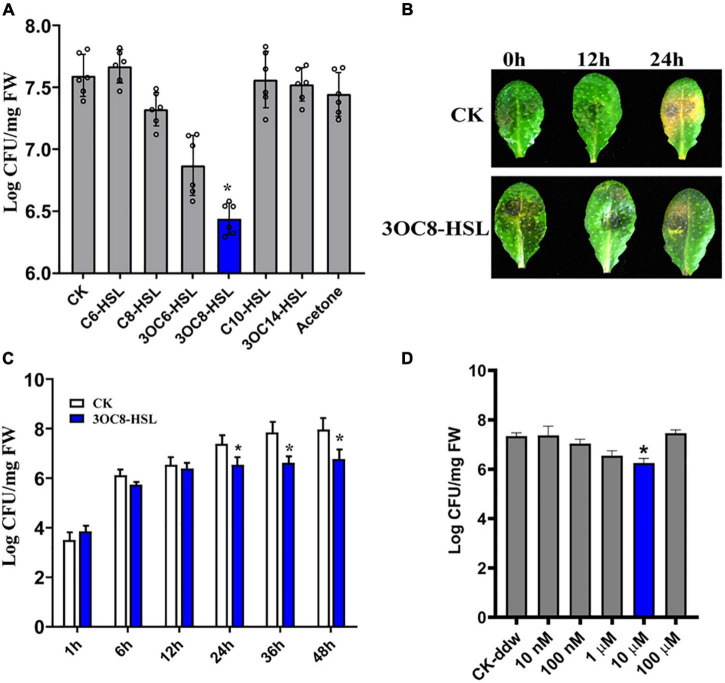
Enhanced resistance of 3OC8-HSL-treated Arabidopsis against *Pcc*. **(A)** Proliferation of *Pcc* in the leaves of Arabidopsis plants (grown in a hydroponic system) in which the roots were pretreated with 10 μM of different AHL compounds for 48 h, and the leaves were subsequently spray-inoculated with 10^7^ CFU/mL *Pcc*. CFUs were counted at 24 h post-inoculation. “CK” is for wild type Arabidopsis *Col-0* inoculated with MgCl_2_. The input of each sample and control were similar, around 10^3^ CFU/g FW. **(B)** Symptoms of *Pcc* infection on wild-type Arabidopsis pretreated with 10 μM 3OC8-HSL. The disease symptoms were recorded at 24 h after inoculation. **(C)** Inhibitory effect of 3OC8-HSL on *Pcc* growth in Arabidopsis. The leaves (grown in hydroponic system) were inoculated with 10^7^ CFU/mL *Pcc* at 48 h after pretreatment with 10 μM 3OC8-HSL at the roots. CFUs were counted at different hour intervals post-inoculation. Data represent the means of three independent biological replicates ± standard deviation (SD). **(D)** Different priming effects of different concentrations of 3OC8-HSL. The experiments were performed with six leaves per treatment, and similar results were obtained in three independent experiments. Asterisks indicate a statistically significant difference between the AHL-pretreated and the water-treated plants (ANOVA test, *P < 0.05). Values are means ± SD of six independent experiments.

*N*-acyl-homoserine lactones confer resistance against biotrophic and hemi-biotrophic pathogens in host plants. However, the effects of AHLs in Arabidopsis against necrotrophic bacteria *Pcc* are still unknown. To evaluate the spectra of AHL-related actions, we pretreated Arabidopsis roots, which were grown in a hydroponic system, for 2 days with six types of AHLs having different chain lengths and modifications at the C3 position. The plant leaves were spray-inoculated with a *Pcc* cell suspension. The bacterial CFUs on the leaf tissues were counted at 24 h post-inoculation. The 3OC8-HSL pretreatment showed the strongest inhibitory effects on pathogen proliferation compared with the unpretreated plants. No significant differences in pathogen propagation were observed in plants pretreated with MgCl_2_, acetone, C6-HSL, C8-HSL, C10-HSL, or 3OC14-HSL (Figure 1A). Then 3OC8-HSL was selected for further analysis. In addition, detached leaves from soil-grown Arabidopsis were pretreated with 10 μM 3OC8-HSL for 2 days prior to spray-inoculation with 10^7^ CFU/mL *Pcc*. The disease symptoms were recorded at 24 h after inoculation. The symptoms of 3OC8-HSL unpretreated leaves having yellow or water-soaked lesions were more serious than those of the 3OC8-HSL pretreated leaves (Figure 1B). To monitor the disease progression on the leaves of 3OC8-HSL-pretreated plants, we determined the CFUs at 48 h after pathogen infection. Pathogen proliferation was significantly inhibited in the 3OC8-HSL-pretreated plants from 24 to 48 h compared with unpretreated plants (Figure 1C). To analyze the effects of AHLs on *Pcc* bacteria, the different concentrations of 3OC8-HSL, ranging from 10 nM to 100 μM, were used as pretreatment and the numbers of *Pcc* CFU were determined. Pathogen proliferation was significantly inhibited in the 10 μM 3OC8-HSL-pretreated plants (Figure 1D).

### Transcriptional Chip Analysis of Arabidopsis Seedlings and Induction of Transcript Factors After Treatment With 3OC8-HSL

To determine the function of 3OC8-HSL in plant-immunity, we used the 29k Arabidopsis Genome Array to profile the gene expressions of Arabidopsis seedlings planted in greenhouse. Seventeen-day-old seedlings were cultured in Hoagland medium with or without 10-μM 3OC8-HSL. Plants were harvested at 24 h after the 3OC8-HSL pretreatment. The transcriptional chip analysis identified a total of 2,589 of the differentially expressed genes (DEGs), including 1,013 upregulated and 1,576 downregulated [National Center for Biotechnology Information (NCBI) Gene Expression Omnibus (GEO) accession number GSE197485]. The data suggested that approximately 9% of the genes were 3OC8-HSL responsive. The DEGs were grouped into 15 functional categories of TAIR. The AHLs were mainly involved in carbohydrate transport and metabolism, signal transduction mechanisms, plant hormone signal transduction, biosynthesis, transport and catabolism of secondary metabolites, defense mechanisms, and large enzyme family mechanisms (Supplementary Figures 1A,B). Notably, ∼90 transcription factors including AP2/ERF-ERF, NAC, WRKY, MYB, C2H2, and bHLH participated in 3OC8-HSL response, and these were similar to those involved in 3OC6-HSL response (Supplementary Table 1). Here, we identified 28 JA- and 13 auxin-related genes responsive to both 3OC8-HSL and 3OC6-HSL.

In molecular function analysis of the microarray data, the expression level-changed transcription factors were listed according to the fold-change value. The expression of *RVE1* (A MYB-like transcription factor that regulates hypocotyl growth by regulating free auxin levels in a time-of-day specific manner) was increased by 26.7 times, the expression of *MYB44* was increased by 3.56 times, and the expression of *WRKY70* was downregulated by 0.44-fold. Several studies have indicated that *AtMYB44* is induced by a number of phytohormones, including abscisic acid, gibberellin acid, JA, ethylene, and auxin. Our previous research suggests that *AtMYB44* may play a role in 3OC6-HSL-mediated primary root elongation by regulating the expressions of auxin- and cytokinin-related genes (Zhao et al., 2016). Our qRT-PCR data showed that *MYB44* responded to 3OC8-HSL, but did not change at the early stage of *Pcc* infection (Supplementary Figure 1C). We also detected the expressions of the genes related to SA synthesis, *ICS1* (encodes isochorismate synthase1), *CBP60g* (encodes calmodulin binding protein 60-like), and *SARD1* (encodes SAR deficient 1), which were involved in 3OC8-HSL response. These results suggested that the SA synthesis seemed to have nothing to do with 3OC8-HSL-induced resistance to *Pcc* (Supplementary Figures 1D-F).

WRKY70 is negatively regulated in the Arabidopsis response to *Pcc* (Li et al., 2017). A WRKY70 deficiency enhances resistance to necrotrophic pathogens by enhancing *PDF1.2* expression through the activation of the JA pathway (Li et al., 2004). Our data suggested that *WRKY70* was significantly reduced after 3OC8-HSL pretreatment and subsequent *Pcc* inoculation (Supplementary Figure 1G). These results suggested that 3OC8-HSL mediated the JA response through WRKY70 and induced disease resistance. RVE1 is a MYB-like transcription factor that regulates hypocotyl growth by regulating free auxin levels, and *RVE1* was elevated after 3OC8-HSL pretreatment (Supplementary Figure 1H). Thus, both JA and auxin pathways may be involved in 3OC8-HSL priming.

### 3OC8-HSL Promotes the Accumulation of Jasmonic Acid

**FIGURE 2:**
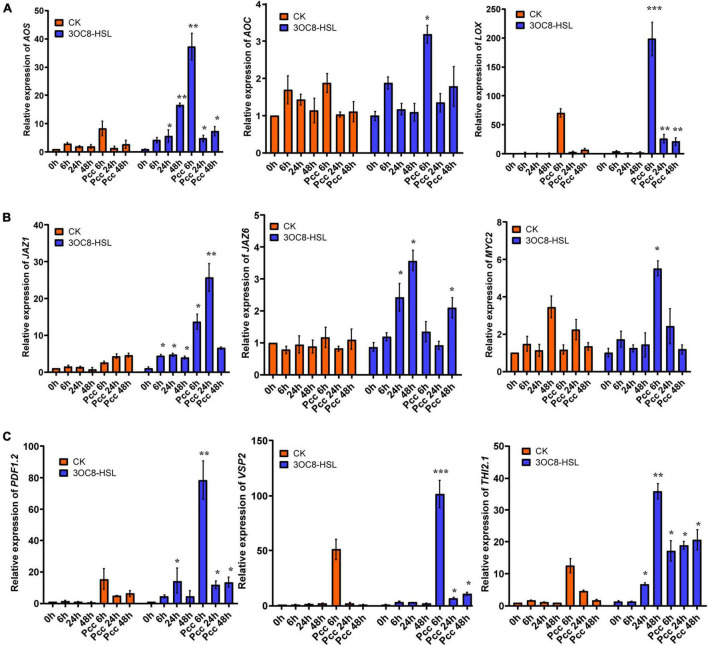
Expressions of the JA-related genes in Arabidopsis plants pretreated with 3OC8-HSL and challenged with *Pcc*. **(A)** Expressions of JA synthesis genes *AOS*, *AOC*, and *LOX*. **(B)** Expressions of JA pathway transcriptional regulators genes *JAZ1*, *JAZ6*, and *MYC2.* **(C)** Expressions of JA downstream cascade genes *PDF1.2*, *VSP2*, and *Thi2.1*. At least five independent experiments were performed, each of which with three technical repeats. Total RNA was extracted from Arabidopsis *Col-0* seedlings pretreated with 10 μM 3OC8-HSL at the roots for 48 h followed by *Pcc* inoculation. Samples were collected at the indicated time points (hpi). Real-time PCR was performed using gene-specific primers, and the relative expression levels of the induced resistance marker genes are shown. Values are means ± SD of five independent experiments. Asterisks indicate a statistically significant difference between the AHL-pretreated and the water-treated plants (ANOVA test, *P < 0.05; **P < 0.01, ***P < 0.001).

To further investigate the effects of 3OC8-HSL on the JA pathway, we monitored the expressions of *LOX* (encodes a lipoxygenase that catalyze the oxygenation of fatty acids), *AOS* (encodes an enzyme that catalyzes the dehydration of the hydroperoxide to an unstable allene oxide in JA biosynthesis), and *AOC* (encodes an enzyme that catalyzes an essential step in JA biosynthesis), which encode key enzymes in the JA biosynthetic pathway (Kazan and Manners, 2013). The pretreatment of roots with 3OC8-HSL resulted in increased expression levels of *LOX*, *AOS*, and *AOC* at 6 h after inoculation with *Pcc* (Figure 2A). Additionally, the expression levels of the JA-signaling marker genes *PDF1.2* (encodes an ethylene- and JA-responsive plant defensin), *VSP2* (has acid phosphatase activity dependent on the presence of divalent cations), and *Thi2.1* (encodes a thionin, which is a cysteine-rich protein having antimicrobial properties) increased dramatically in the leaves of plants primed with 3OC8-HSL when compared with unpretreated plants. The induction of gene expressions by 3OC8-HSL reached a maximum at 6 h after *Pcc* infection. MYC2 (encodes a MYC-related transcriptional activator with a typical DNA binding domain of a basic helix-loop-helix leucine zipper motif), the transcription factor of JA-response genes, was crucial to the JA-signaling pathway. JAZs (encodes jasmonate-zim-domain protein) bind to MYC2 and inhibit their dissociation. Transcriptional levels of *MYC2*, *JAZ1*, and *JAZ6* were also examined. After AHL-pretreatments, the expression of *MYC2* increased 5.8 times at 6 h after inoculation compared with the unpretreated plants. Additionally, the expression of *JAZ1* increased rapidly to a 30-fold increase at 6 h after inoculation. *JAZ6* expression also increased, but the changes were not greater than those of *JAZ1* (Figures 2B,C). This implied that JA signaling is involved in 3OC8-HSL priming in Arabidopsis.

**FIGURE 3:**
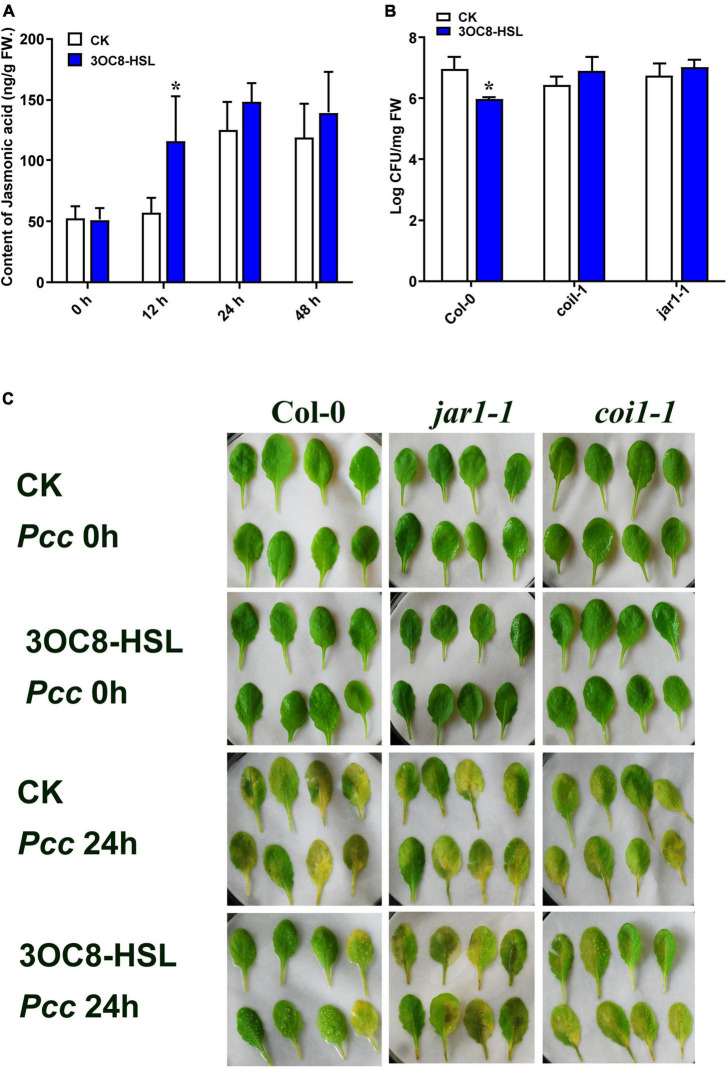
Effects of 3OC8-HSL application on the accumulation of JA and effects of 3OC8-HSL on *Pcc* growth in wild-type Arabidopsis *Col-0*, *coi1-1*, and *jar1-1*. **(A)** Accumulation of free JA measured by HPLC in Arabidopsis plants in which the roots were pretreated with 10 μM 3OC8-HSL for 48 h and the leaves were subsequently spray-inoculated with 10^7^ CFU/mL *Pcc*. **(B)** Proliferation of *Pcc* in the leaves of *Col-0*, *coi1-1*, and *jar1-1*. **(C)** Symptoms of *Pcc* infection *Col-0*, *coi1-1*, and *jar1-1*. At least five independent experiments were performed, each of which with three technical repeats. Asterisks indicate a statistically significant difference between the AHL-pretreated and the water-treated plants (ANOVA test, *P < 0.05). Values are means ± SD of six independent experiments.

To further explore the role of JA in 3OC8-HSL-treated plants, the content of JA was detected by HPLC using the internal standard method. Plant roots were pretreated with 3OC8-HSL for 48 h. Afterward, the JA content was slightly induced, whereas further inoculations of *Pcc* dramatically promoted JA and JA-Ile accumulations in leaves (Figure 3A and Supplementary Figure 2). The results indicated that 3OC8-HSL was primarily involved in plants responding against *Pcc* by quickly elevating the accumulation of JA, which was in agreement with the enhanced accumulation of JA at this time point after the subsequent inoculation of *Pcc* (Figure 3A).

### The Priming Effect Is Absent in JA-Perception Defective Mutant *coi1-1* and *jar1-1*

**FIGURE 4:**
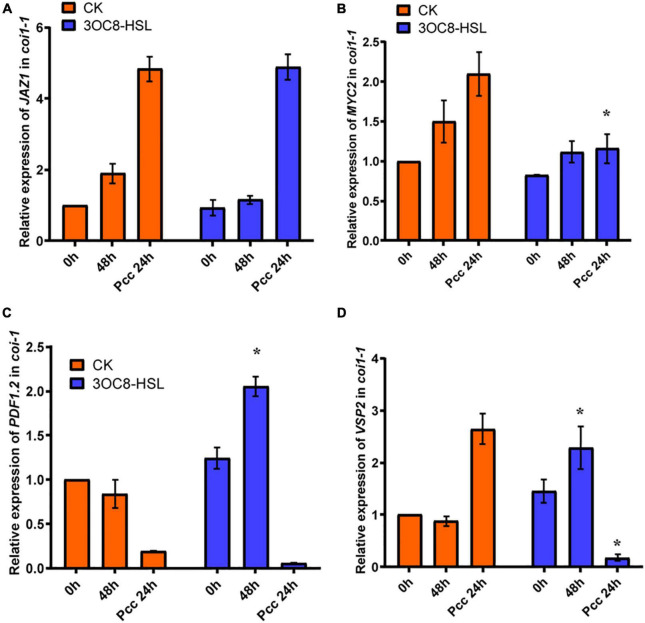
Expressions of JA regulatory genes and response genes in JA pathway mutant *coi1-1*. **(A)** Expression of *JAZ1* in *coi1-1* after 3OC8-HSL pretreated or not. **(B)** Expression of *MYC2* in *coi1-1* after 3OC8-HSL pretreated or not. **(C)** Expression of *PDF1.2* in *coi1-1* after 3OC8-HSL pretreated or not. **(D)** *VSP2* in *coi1-1* after 3OC8-HSL pretreated or not. At least five independent experiments were performed, each of which with three technical repeats. Asterisks indicate a statistically significant difference between the AHL-pretreated and the water-treated plants (ANOVA test, *P < 0.05). Values are means ± SD of five independent experiments.

To further investigate the role of the JA-defense signaling cascade in 3OC8-HSL-induced plants, we compared the resistance of wild-type Arabidopsis plants (*Col-0*) and mutants impaired in the JA-signaling pathway. The *jar1-1* mutant is defective in the synthesis of JA-Ile, the active compound in JA signaling (Chini et al., 2007), whereas *coi1-1* is defective in JA perception (Yang et al., 2019). Unlike wild-type plants, which showed significantly reduced bacterial proliferation levels after pretreatment with 3OC8-HSL, the *jar1-1* and *coi1-1* mutants exhibited no difference in *Pcc* proliferation levels, independent of the 3OC8-HSL pretreatment, which suggested that the 3OC8-HSL-induced resistance required JAR1 and COI1 (Figures 3B,C). Thus, our findings demonstrated that the perception or the synthesis of the active JA-Ile was required for the 3OC8-HSL-primed resistance to *Pcc* in Arabidopsis. We also examined the expression levels of the JA transcriptional regulators *JAZ1* and *MYC2*, as well as the response genes *PDF1.2* and *VSP2* in the *coi1-1* mutant. In *COI1*-deficient mutant, the *JAZ1* expression level was the same as in *Col-0*. In addition, the expression of *MYC2*, which increased four-fold in *Col-0*, showed no changes in *coi1-1* mutant (Figures 4A,B). Downstream marker genes *PDF1.2* and *VSP2* also increased significantly, suggesting that the 3OC8-HSL priming of *Pcc* resistance was dependent on JA-Ile perception (Figures 4C,D).

### The Auxin Pathway Is Involved in 3OC8-HSL Priming

**FIGURE 5:**
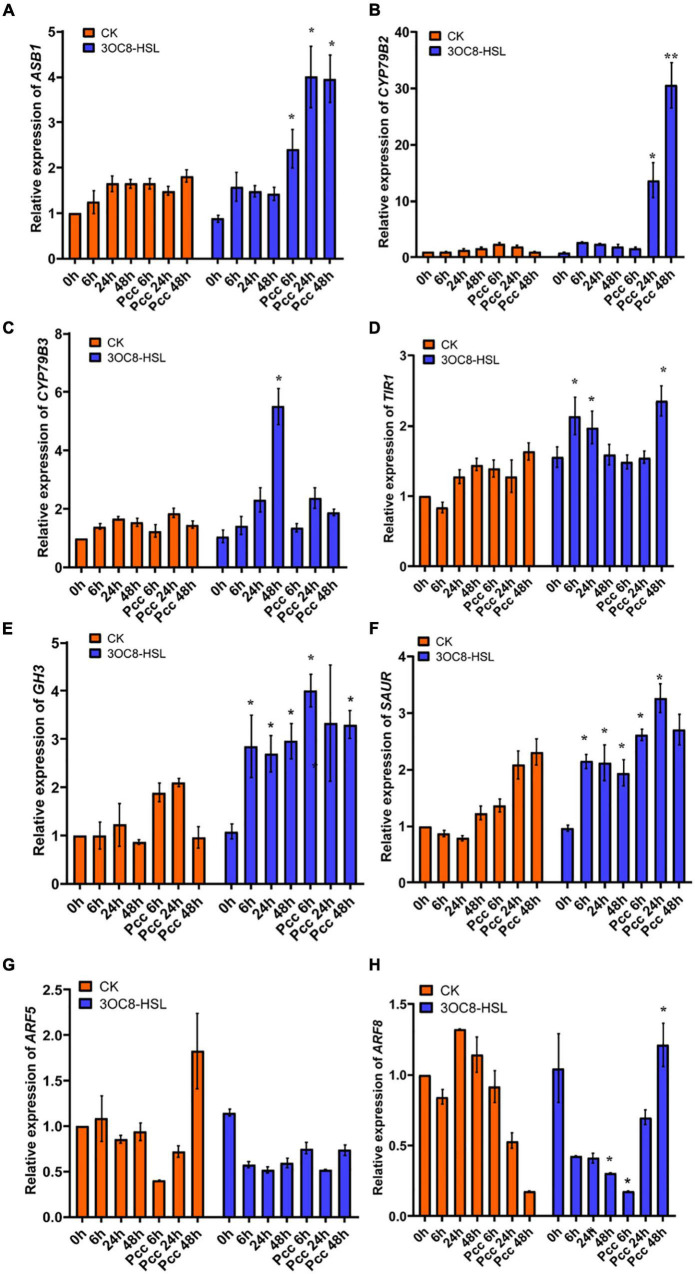
Expressions of the auxin-related genes in Arabidopsis plants pretreated with 3OC8-HSL and challenged with *Pcc*. **(A)** Expression of Auxin pathway transcriptional regulators genes *ASB1*. **(B)** Expression of auxin pathway transcriptional regulators gene *CYP79B2.* **(C)** Expression of auxin perception gene *CYP79B3.* **(D)** Expression of auxin synthesis gene *TIR1.* **(E)** Expression of auxin synthesis gene *GH3.* **(F)** Expression of auxin synthesis gene *SAUR*. **(G)** Expression of auxin downstream cascade gene *ARF5*. **(H)** Expression of auxin downstream cascade gene *ARF7*. At least five independent experiments were performed, each of which with three technical repeats. Asterisks indicate a statistically significant difference between the AHL-pretreated and the water-treated plants (ANOVA test, *P < 0.05). Values are means ± SD of five independent experiments.

In accordance with the gene chip data, we verified the upregulation of auxin-related transcription factors by 3OC8-HSL using qRT-PCR. We pretreated *Col-0* plants with 3OC8-HSL for 48 h and detected the genes’ expression levels at 6, 24, and 48 h after inoculation. 3OC8-HSL-pretreated seedlings showed substantially increased transcript levels of the auxin biosynthetic genes *ASB1* (encodes an anthranilate synthase beta subunit 1), *CYP79B2* (encodes a cytochrome P450, family 79, subfamily B, polypeptide 2), and *CYP79B3* (encodes a cytochrome P450, family 79, subfamily B, polypeptide 3) after inoculation (Figures 5A-C). We also analyzed the expression pattern of several genes involved in auxin perception and transport, as well as transcription factors after exposure to 3OC8-HSL. As shown in Figures 5D-F, after *Col-0* plants were treated with 3OC8-HSL, the expression levels of *TIR1* (encodes an auxin receptor that mediates auxin-regulated transcription), *GH3* (encodes an IAA-amido synthase that conjugates Asp and other amino acids to auxin *in vitro*), and *SAUR* (encodes a small auxin-up RNA) were basically stable. However, after inoculation with *Pcc*, the expressions of *TIR1*, *GH3*, and *SAUR* increased within 48 h in all the AHL-pretreated seedlings (Figures 5D-F).

Auxin response factors (ARFs) are transcriptional factors that bind to the specific DNA sequence 5’-TGTCTC-3’ found in auxin-responsive promoter elements. ARF5 mediates embryo axis formation and vascular tissues’ differentiation (Spaepen and Vanderleyden, 2011), and it did not dramatically change in AHL-pretreated plants compared with the unpretreated plants (Figure 5G). ARF8 has been reported to be required for JA biosynthesis (Werghi et al., 2021), and it had a lower expression level after unpretreated plants were inoculated with *Pcc*. The downregulation of *ARF8* leads to a decrease in auxin responses and JA synthesis (Yang et al., 2019). However, AHL pretreatment prior to inoculation with *Pcc* rescued the repression of *ARF8* in plants, resulting in increased resistance to *Pcc* (Figure 5H).

**FIGURE 6:**
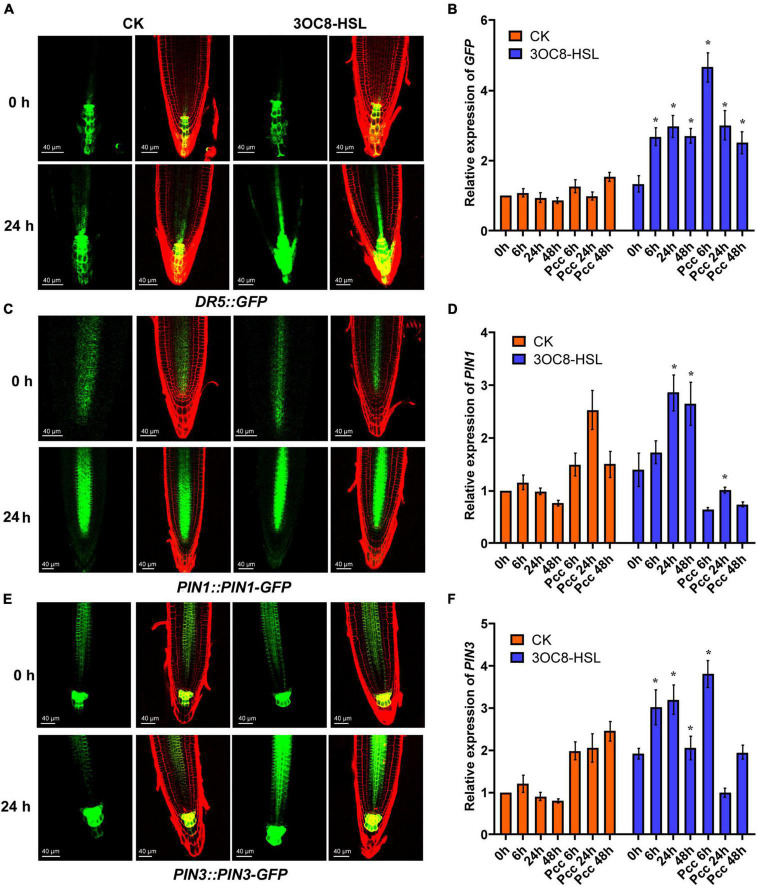
AHL-induced expressions of auxin response gene *DR5*, auxin transport gene *PIN1* and *PIN3*. **(A,C,E)** GFP fluorescence of primary roots (PR), in 5-d-old *DR5::GFP*, *PIN1::PIN1-GFP, PIN3::PIN3-GFP* seedlings exposed to 10 μM 3OC8-HSL for 24h. *DR5::GFP* auxin response is increased in QC. Cell walls were stained with PI. We performed the experiment 5 times and 30 roots each treatment. The left panels show the GFP fluorescence, on the right is the merged image of PI stained cell wall with GFP fluorescence. **(B,D,F)** Expressions of *GFP*, *PIN1*, *PIN3.* At least five independent experiments were performed, each of which with three technical repeats. Asterisks indicate a statistically significant difference between the AHL-pretreated and the water-treated plants (ANOVA test, *P < 0.05). Values are means ± SD of five independent experiments.

To investigate the influence of auxin on priming effect initiated by 3OC8-HSL, we measured the expression of the GFP reporter gene in *DR5::GFP* (the auxin-response marker) transgenic Arabidopsis. The results showed that the intensity of GFP fluorescence in primary root cells increased at 24 h after 10-μM 3OC8-HSL pretreatment (Figure 6A). These results indicate that 3OC8-HSL induced the accumulation of auxin in the primary root tips of Arabidopsis. The GFP expression level was significantly induced in seedlings by AHL pretreatment and increased more sharply after inoculation with *Pcc* bacteria, reaching five times the level in the unpretreated plants (Figure 6B). Similarly, the 10 μM 3OC8-HSL treatment induced a rapid increase in the GUS activity in *DR5::GUS* transgenic plants (Supplementary Figure 3).

**FIGURE 7:**
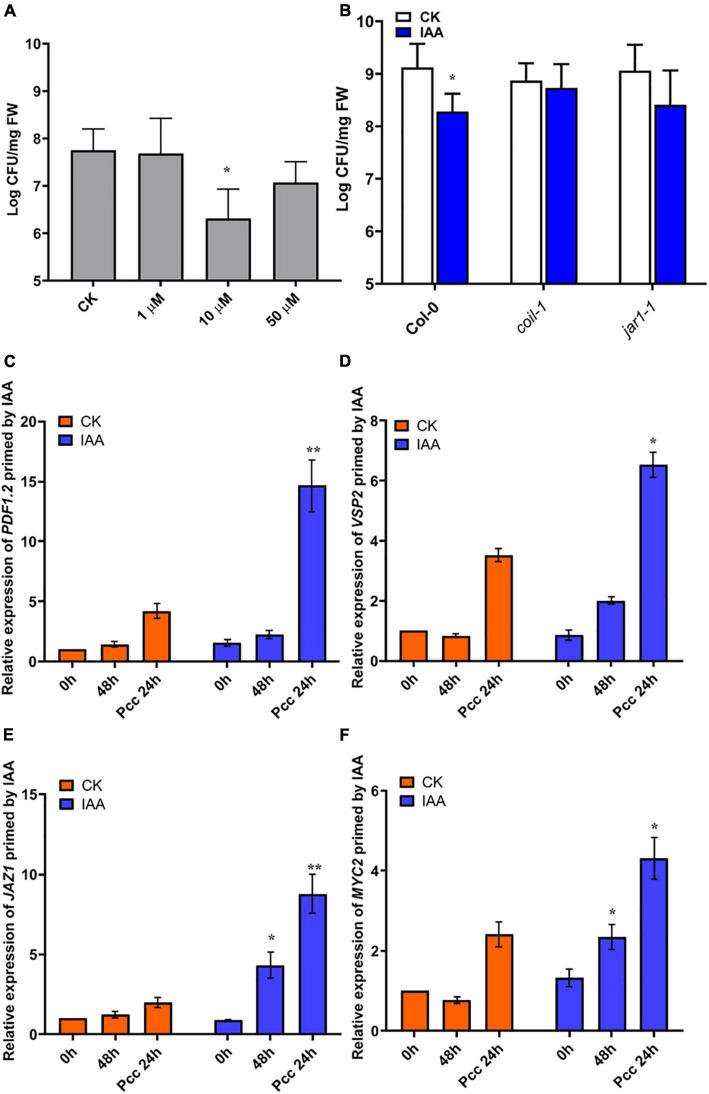
IAA induces resistance to *Pcc* in a JA-dependent manner. **(A)** Effects of different concentrations of IAA on the proliferation of *Pcc* in the leaves of Arabidopsis plants (grown in a hydroponic system). **(B)** Proliferation of *Pcc* in the leaves of *Col-0*, *coi1-1*, *jar1-1*(grown in a hydroponic system) in which the roots were pretreated with 10 μM IAA for 48 h and the leaves were subsequently spray-inoculated with 10^7^ CFU/mL *Pcc*. **(C–F)** Expressions of *JAZ1*, *MYC2*, *PDF1.2*, and *VSP2* induced by 10 μM IAA. At least five independent experiments were performed, each of which with three technical repeats. Asterisks indicate a statistically significant difference between the AHL-pretreated and the water-treated plants ANOVA test, (*P < 0.05, **P < 0.01). Values are means ± SD of five independent experiments.

The PIN proteins are important regulators involved in the establishment of the auxin gradient and the maximum auxin level in the root apex. We used the *PIN1::PIN1-GFP* and *PIN3::PIN3-GFP* transgenic lines to monitor their expression levels. As shown in Figure 6, the 3OC8-HSL pretreatment significantly promoted the expression level of *PIN3*, but not *PIN1*, compared with in the unpretreated plants (Figures 7B-D). These data indicated that *PIN3* may involve in the 3OC8-HSL-induced changes in auxin accumulation and distribution in plant tissues.

### 3OC8-HSL-Primed IAA-Mediated Resistance to *Pcc* Depends on the JA Pathway

Our microarray data suggested that significant changes in auxin signaling pathway-related genes occurred after 3OC8-HSL pretreatment. Consequently, we measured plant resistance to *Pcc* after roots pretreatment with 10 μM IAA. We found that IAA significantly reduced *Pcc* count numbers in pretreated plants (Figure 7A). We also found that the IAA pretreatment could induce elevation in the expression levels of JA pathway genes, such as *JAZ1*, *MYC2*, *PDF1.2*, and *VSP2*. Thus, the JA pathway was activated by the IAA pretreatment. We detected bacterial cell numbers in *coi1-1* and *jar1-1* mutants after the IAA pretreatment, and the *Pcc* CFU was similar in the IAA unpretreated mutants (Figure 7B). This indicated that IAA-induced resistance to *Pcc* was dependent on the JA pathway. Because 3OC8-HSL can induce the JA and auxin pathways, the priming effect may result from the integration of the JA and auxin pathways.

### 3OC8-HSL Promotes Resistance Against *Pcc* in Chinese Cabbage

**FIGURE 8:**
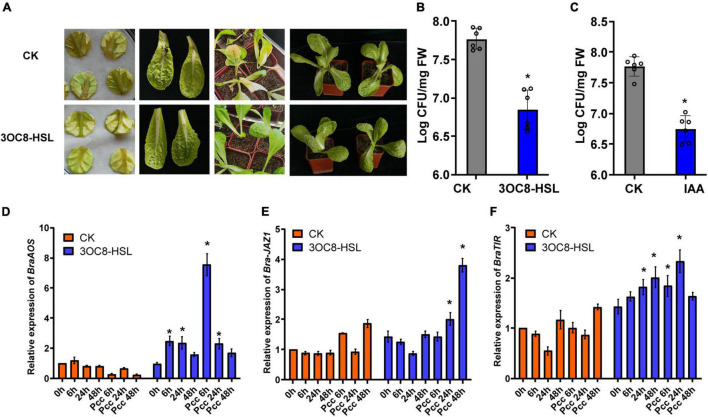
**(A)** Symptoms of *Pcc* infection in Chinese cabbage. The top line is untreated Chinese cabbage leaves inoculated with *Pcc*, and the bottom line is 3OC8 pretreated leaves inoculated with *Pcc.* **(B)** AHL priming effect in Chinese cabbage. The proliferation of *Pcc* in the leaves of Chinese cabbage (grown in pot) in which the roots were pretreated with 10 μM 3OC8-HSL for 48 h and the leaves were subsequently spray-inoculated with 10^7^ CFU/mL *Pcc*. **(C)** IAA priming effect in Chinese cabbage. The proliferation of *Pcc* in the leaves of Chinese cabbage (grown in pot) in which the roots were pretreated with 10 μM IAA compound for 48 h and the leaves were subsequently spray-inoculated with 10^7^ CFU/mL *Pcc*. **(D–F)** Expressions of *BraAOS*, *BraJAZ1*, and *BraTIR1* induced by 10 μM IAA. At least five independent experiments were performed, each of which with three technical repeats. Asterisks indicate a statistically significant difference between the AHL-pretreated and the water-treated plants (ANOVA test, *P < 0.05). Values are means ± SD of five independent experiments.

To explore the application effects of 3OC8-HSL on Chinese cabbage resistance, detached leaves and potted plants were used. The lesion areas of the leaves treated with 3OC8-HSL were significantly smaller than those on leaves of plants not receiving AHL treatment. For the potted plants, the main veins of leaves from unpretreated plants were broken and slowly withered owing to the decay at the inoculation site (Figure 8A). The 3OC8-HSL pretreatment significantly reduced *Pcc* colonization in Chinese cabbage leaves (Figure 8B). Like 3OC8-HSL, IAA had a priming effect on *Pcc*, resulting in the reduced bacterial colonization of Chinese cabbage. In addition, the JA synthesis gene *BraAOS (Bra035320*), auxin-related gene *BraTIR1*, and *BraJAZ1* in Chinese cabbage were detected. The expression levels of *BraAOS*, *BraJAZ1*, and *BraTIR1* were significantly increased after AHL pretreatment (Figures 8D-F).

## Discussion

Priming is defined as an induced state whereby a plant reacts more rapidly and more efficiently to stress. This is an adaptive strategy that improves the defensive capacity of plants (Tugizimana et al., 2018). The primed plants may respond to very low levels of stimuli in a faster and stronger manner than unprimed plants (Mauch-Mani et al., 2017). In this study, we demonstrated that 3OC8-HSL pretreated plants acted as an inducer of resistance against the necrotrophic pathogen *Pcc* in Arabidopsis (Figure 1). We found that 3OC8-HSL pretreatment did not affect the JA content and JA-related genes’ expression levels, whereas those of the auxin-response gene *DR5* were slightly induced (Figures 2, 3, 6). After *Pcc* inoculation, there were dramatically induced expression levels of both auxin and JA pathways (Figures 2–5). These phenomena indicated that the 3OC8-HSL pretreatment only primed the plants to maintain a ready-to-go state that had no or minimal negative impacts on the host plants’ energy status. When encountering stresses, such as *Pcc* invasion, the primed plants defended against the bacterial infection more rapidly and dramatically than unprimed plants (Figure 8). Thus, we reported that the primed plants are more sensitive to stresses involved in induced systemic resistance and systemic acquired resistance activation (Katagiri, 2018). Consistent with these findings, the root pretreatment of the plants with 3OC8-HSL led to the upper leaves (distal tissues) being more resistant to the bacterial pathogen (Figure 7), but 3OC8-HSL was not detected in these leaves, which indicated that 3OC8-HSL triggers plant immunity by activating induced systemic resistance or systemic acquired resistance.

Numerous AHLs have been identified from 70 species of Gram-negative bacteria. It was reported that AHLs with short side chains (four to six carbons) regulate root growth and development, whereas long-chain AHLs such as C12- and C14-HSL induce plant resistance (Schenk and Schikora, 2014). In our study, we showed that 3OC8-HSL was a short-chain AHL that enhanced resistance against the necrotrophic pathogen *Pcc* in Arabidopsis and cabbage. The phenomena were similar to those found in 3OC14-HSL-pretreated plants, in which triggered defense does not respond. The 3OC14-HSL-pretreated plants showed faster and stronger activation of defense responses than unpretreated plants after inoculation with biotrophic and hemi-biotrophic pathogenic bacteria or a pathogenic elicitor flagellin peptide (flg22, Schenk et al., 2014). 3OC6-HSL also induces the priming of plants, which indicated that AHLs carbonylated at the C3-position of fatty acid side chains might play an important role in regulating plant disease resistance.

Our previous data indicate that 3OC8-HSL primes the Arabidopsis defense response to hemi-biotrophic bacterial infection and that 3OC8-HSL-primed resistance is dependent on the SA-signaling pathway (Liu et al., 2020). Cross-talk between the defense signaling hormones SA and JA, as well as growth regulators play significant roles in mediating the trade-off between growth and defense in plants. Recent studies have provided new insights into the role of auxin in plant defense. Although SA does not affect auxin biosynthesis, it represses the expressions of auxin receptor genes *TIR1/AFBs* to reduce the auxin response (Navarro et al., 2006; Wang et al., 2007). In contrast to the well-recognized antagonistic crosstalk between SA and auxin during plant resistance to biotrophic pathogens, Qi et al. (2012) demonstrated that *Alternaria brassicicola* infection led to an enhanced auxin response in host plants. This also provided molecular evidence that supported the hypothesis in which JA and auxin promoted plant resistance to necrotrophic pathogen coordinately. When plants are pretreated by exogenous auxin, the auxin–TIR–AUX/IAA–ARF signaling is activated, then JA synthesis increases, which leads to further up-regulation of auxin synthase genes and the auxin accumulation.

The plant hormone JA plays essential roles in many biological processes including plant defense against necrotrophic pathogens (Kazan and Manners, 2013). We found that the JA pathway was not affected by AHLs treatment, and those significant changes occurred after the plants were inoculated with the necrotrophic pathogen *Pcc*. The JA perception impaired mutant *coi1-1* and JA synthesis impaired mutant *jar1-1*, did not show the priming effects. These results indicated that the JA pathway was essential in mediating resistance to necrotrophic pathogens. The rapidly increasing expression levels of JA-responsive genes, such as *PDF1.2* and *VSP2*, in 3OC8-HSL-pretreated plants may contribute to the elevated response of primed plants. The 3OC8-HSL pretreatment also induced the expressions of auxin pathway genes, and consequently, IAA priming effects played the same roles in the resistance against *Pcc*, but the effects were absent in the *coi1-1* or *jar1-1* mutant, suggesting that IAA-induced resistance was dependent on the JA pathway. Consistently, Qi et al. (2012) demonstrated that JA and auxin interact by positively regulating plant resistance to the necrotrophic pathogen *A. brassicicola*, and that auxin signaling is activated through JA, which may contribute to plant resistance to necrotrophic pathogens. We deduced that the synergistic induction of JA and auxin, as well as the underlying molecular mechanism need to be further explored. These findings will expand our understanding of the mechanisms that plants use to respond to 3OC8-HSL and will provide insights into novel applications of these biological molecules in regulating crop growth and development.

## Data Availability Statement

The datasets presented in this study can be found in online repositories. The names of the repository/repositories and accession number(s) can be found below: https://www.ncbi.nlm.nih.gov/, GSE197485.

## Author Contributions

FL and QZ performed the plant pathology analysis and molecular, genetic assay, respectively. ZJ performed the network analysis. SZ and JW helped with the acquisition, analysis, and interpretation of data for the work. YJ and SS were responsible for the design of the work. All authors gave approval to the final version.

## Conflict of Interest

The authors declare that the research was conducted in the absence of any commercial or financial relationships that could be construed as a potential conflict of interest.

## Publisher’s Note

All claims expressed in this article are solely those of the authors and do not necessarily represent those of their affiliated organizations, or those of the publisher, the editors and the reviewers. Any product that may be evaluated in this article, or claim that may be made by its manufacturer, is not guaranteed or endorsed by the publisher.

## Footnotes

1
http://www.arabidopsis.org

## Supplementary Material

Supplementary Figure 1 Enrichment analysis of differential genes expressions in microarray and expressions of key transcription factors. **(A)** GO classification plots and enrichment scatter plots of differentially expressed genes. The larger the enrichment factor indicates the more significant the enrichment level of differentially expressed genes in this pathway, the size of the circle indicates the number of genes enriched in the pathway, and the larger the circle indicates the more genes. **(B)** Pathway classification and enrichment of differentially expressed genes. **(A)** and **(B)** were plotted by http://www.bioinformatics.com.cn, a free online platform for data analysis and visualization. **(C)** Expression of the transcription factor gene *MYB44*. **(D)** Expression of the SA synthesis related gene *ICS1*. **(E)** Expression of the SA synthesis related gene *SARD1*. **(F)** Expression of the SA synthesis related gene *CBP60g*. **(G)** Expression of the transcription factor gene *WRKY70*. **(H)** Expression of the transcription factor gene *RVE1*. For genes’ expressions, Arabidopsis plants were pretreated with 3OC8-HSL and challenged with *Pcc*. The experiments were performed at least five independent experiments with three technical repeats. Asterisks indicate a statistically significant difference between the AHL-pretreated and the water-treated plants (ANOVA test, **P* < 0.05). Values are means ± SD of five independent experiments.

Supplementary Figure 2 Effects of 3OC8-HSL application on the accumulation of JA-Ile. Accumulation of JA-Ile was measured by ELISA in Arabidopsis plants in which the roots were pretreated with 10 μM 3OC8-HSL for 48 h and the leaves were subsequently spray-inoculated with 10^7^ CFU/mL *Pcc*. The experiments were performed at least three independent experiments with three technical repeats. Asterisks indicate a statistically significant difference between the AHL-pretreated and the water-treated plants (ANOVA test, **P* < 0.05). Values are means ± SD of three independent experiments.

Supplementary Figure 3 AHL induced expression GUS in *DR5::GUS* plant. The experiments were performed at least five independent experiments with three technical repeats. Asterisks indicate a statistically significant difference between the AHL-pretreated and the water-treated plants (ANOVA test, **P* < 0.05). Values are means ± SD of five independent experiments.
