# Supplementary figures and images for: N-3-Oxo-Octanoyl Homoserine Lactone Primes Plant Resistance Against Necrotrophic Pathogen Pectobacterium carotovorum by Coordinating Jasmonic Acid and Auxin-Signaling Pathways
Source: Front Plant Sci. 2022 Jun 14;13:886268. doi: 10.3389/fpls.2022.886268 (PMC9237615; doi:10.3389/fpls.2022.886268)

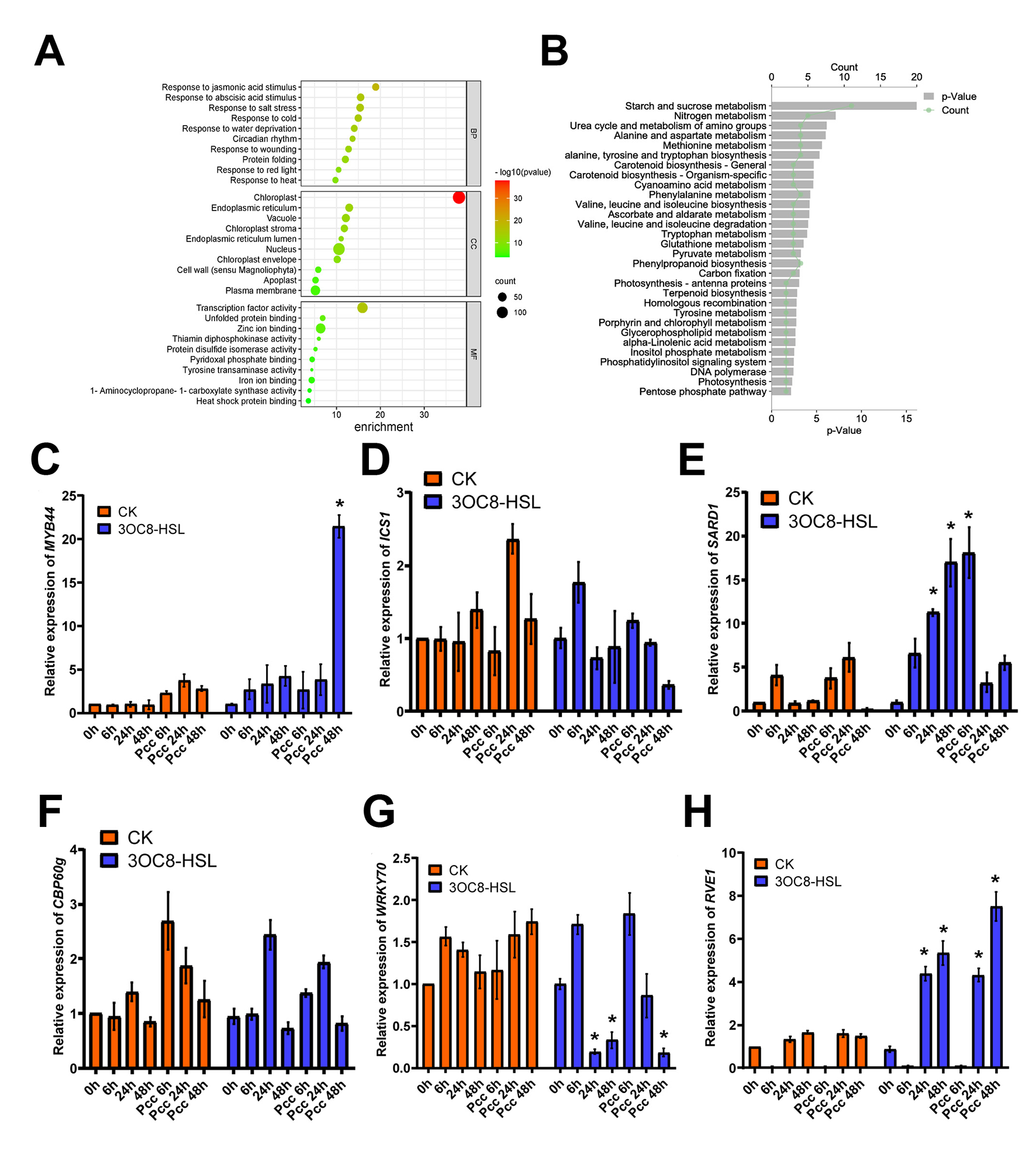

Supplement: Supplementary Figure 1 — Enrichment analysis of differential genes expressions in microarray and expressions of key transcription factors. (A) GO classification plots and enrichment scatter plots of differentially expressed genes. The larger the enrichment factor indicates the more significant the enrichment level of differentially expressed genes in this pathway, the size of the circle indicates the number of genes enriched in the pathway, and the larger the circle indicates the more genes. (B) Pathway classification and enrichment of differentially expressed genes. (A) and (B) were plotted by http://www.bioinformatics.com.cn, a free online platform for data analysis and visualization. (C) Expression of the transcription factor gene MYB44. (D) Expression of the SA synthesis related gene ICS1. (E) Expression of the SA synthesis related gene SARD1. (F) Expression of the SA synthesis related gene CBP60g. (G) Expression of the transcription factor gene WRKY70. (H) Expression of the transcription factor gene RVE1. For genes’ expressions, Arabidopsis plants were pretreated with 3OC8-HSL and challenged with Pcc. The experiments were performed at least five independent experiments with three technical repeats. Asterisks indicate a statistically significant difference between the AHL-pretreated and the water-treated plants (ANOVA test, *P < 0.05). Values are means ± SD of five independent experiments. [file Image_1.TIF]

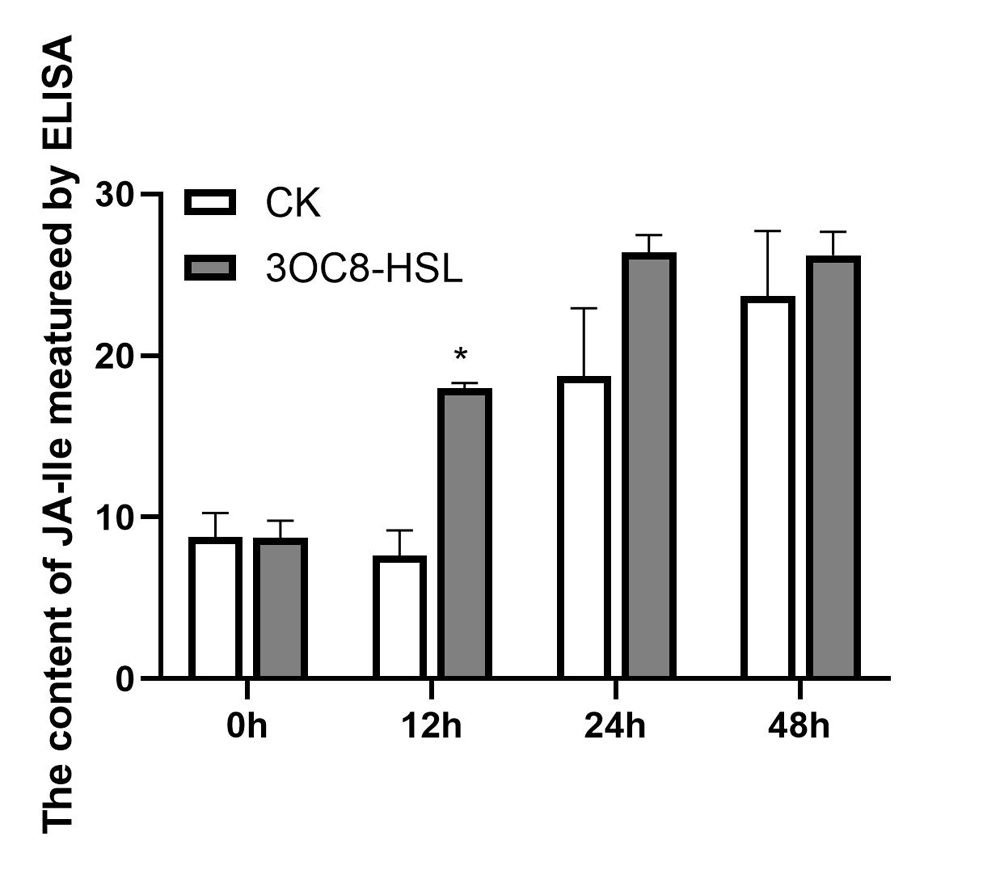

Supplement: Supplementary Figure 2 — Effects of 3OC8-HSL application on the accumulation of JA-Ile. Accumulation of JA-Ile was measured by ELISA in Arabidopsis plants in which the roots were pretreated with 10 μM 3OC8-HSL for 48 h and the leaves were subsequently spray-inoculated with 107 CFU/mL Pcc. The experiments were performed at least three independent experiments with three technical repeats. Asterisks indicate a statistically significant difference between the AHL-pretreated and the water-treated plants (ANOVA test, *P < 0.05). Values are means ± SD of three independent experiments. [file Image_2.TIF]

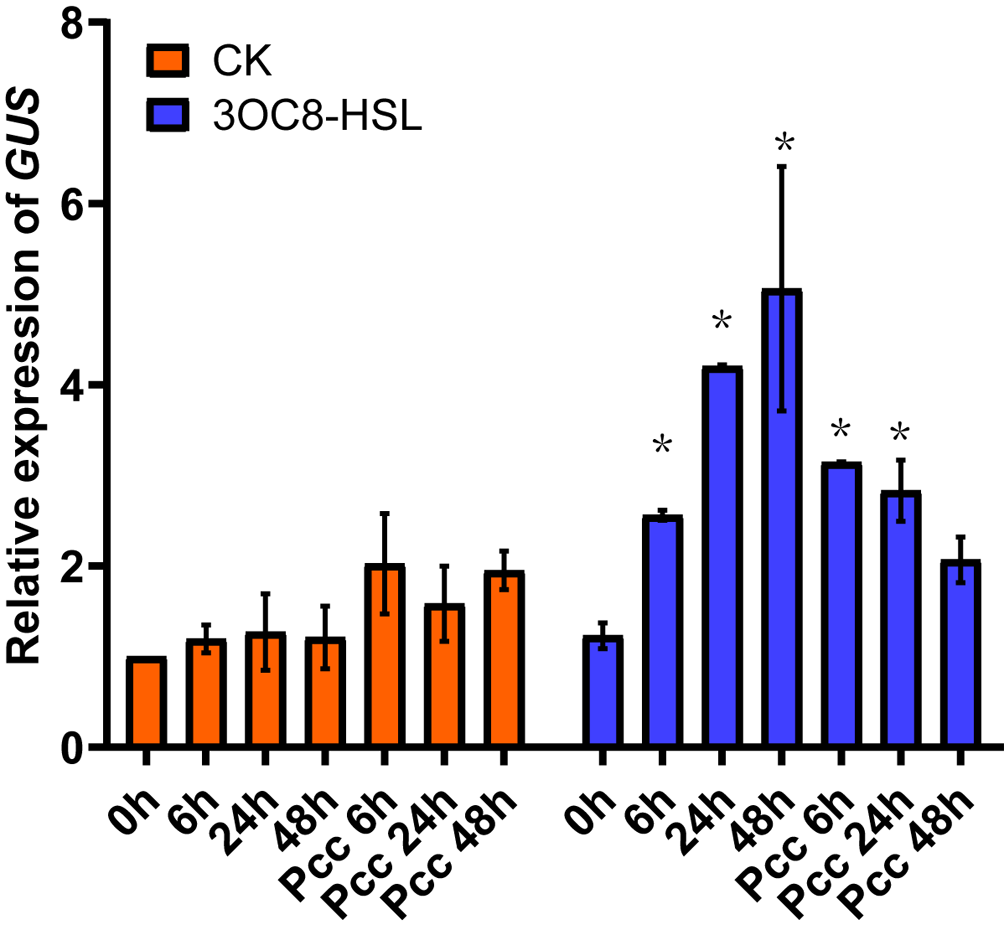

Supplement: Supplementary Figure 3 — AHL induced expression GUS in DR5::GUS plant. The experiments were performed at least five independent experiments with three technical repeats. Asterisks indicate a statistically significant difference between the AHL-pretreated and the water-treated plants (ANOVA test, *P < 0.05). Values are means ± SD of five independent experiments. [file Image_3.TIF]
