# Supplementary material for: N-3-Oxo-Octanoyl Homoserine Lactone Primes Plant Resistance Against Necrotrophic Pathogen Pectobacterium carotovorum by Coordinating Jasmonic Acid and Auxin-Signaling Pathways
Source: Front Plant Sci. 2022 Jun 14;13:886268. doi: 10.3389/fpls.2022.886268 (PMC9237615; doi:10.3389/fpls.2022.886268)
Supplement: Supplementary file 4 [file Table_1.DOC]

**Supplementary Table S1 Primer information of genes investigated in qRT-PCR**

qRT-PCR was done using primers listed in the table.

| Primer | Sense | Antisense |
| --- | --- | --- |
| *ICS1* | GAACTCAAATCTCAACCTCC- | ACTGCGACGAG AGAAGAAAC |
| *SARD1* | GGAATGTCTGATAGAAAGTGGGAAGT | ACGTAGGGCTGGTTGAGGGA |
| *CBP60g* | GTTCTCGTCTTCTCGGGTCG | CATCACCGTTAGGTCTCCAGT |
| *Actin2/8* | CCAGAAGGATGCATATGTTGGTGA | GAGGAGCCTCGGTAAGAAGA |
| *TIR1* | GCCTTTTGTCATGGAACCAAAT | ATTTGACGGCAGAAGTAGAGAA |
| *ARF8* | GCTATCGAAGGGTTGTTGGAA | CTCCATGATCTCCCATTTGAT |
| *ARF5* | GATAATTCTGGAGGCACTGGTA | CAAACATGCATTCGATAGCAGA |
| *GUS* | GTCGCGCAAGACTGTAACCA | CGGCGAAATTCCATACCTG |
| *GFP* | CGTCTATATCATGGCCGACAAGCA | TCGCCGATGGGGGTGTTCTG |
| *PIN3* | GAGGGAGAAGGAAGAAAGGGAAAC | CTTGGCTTGTAATGTTGGCATCAG |
| *JAZ6* | AAAAGCAACGTGGTGATTCC | CCTGTCTTTTCGTTTAGCAAAGA |
| *SAUR* | TGGCTTTGGTGAGAAGTCTATT | TACTGAGCAGAGCTTGAAAAGA |
| *ASB* | GGGGAAGAGTCGTAGAGATGTCT | CTGGCAGAGATTGTATGTGAAGC |
| *CYP79B3* | AGTCACTTCCGAACACTCA | TCGCAGGTTACCATATTCC |
| *CYP79B2* | CACGATGATGCTCGCGAGACT | TCACTTCACCGTCGGGTAGAGA |
| *MYB44* | TAGCCACGAGTCAACGAATATCAAC | CAATCGCACCTCTAAACCCACC |
| *At5g17300* | AAGCAACTGGAGGTGATGGGAGC | TGGGTGAACTCGAATCAAGGGAA |
| *WRKY70* | TCAGCAACTCCTCTCTCAACCCG | GGAATCTTCAAACTTGCCGTCGT |
| *JAZ1* | CTTTTATGCCGGTTCAGTTTGTGTT | GCTTTTGTGGCTCCGAGTCCGTTTG |
| *AOS* | TCCGACGGTGGGGAATAAACAATG | AACAGATGGACTACACAGGTGCGAAC |
| *AOC* | TCGTAAGCGTAATGTGTCCCGTCCCT | CGAGCCTACTAAGCCAAACTTTCCAA |
| *LOX2* | CAGAGCAACGCTACGGGGGAGAG | AGAACTGGGGCATCAAACTGGAGAAT |
| *PDF1.2* | CATGGCTAAGTTTGCTTCCA | GTTGCATGATCCATGTTTGG |
| *MYC2* | TTGATGAGGAGGTGACGGATAC | CCAAACACTCCTCCTTGCTTAG |
| *VSP2* | AGCATCTCATACTCAAGCCAAACG | AGTATCCTCAACCAAATCAGCCCA |
| *GH3* | CCATCTCTGAGTTCCTCACAAGC | TCCTCTTCGATTGTTGGCATTAGC |
| *PIN1* | TGGAAGACAACCTTTGGAAACT | TGAAGCATTAGAACGACGAACA |
| *BraAOS* | CCTAGTCGCAAGCGGTTGAT | AGGGATTGAGCAGATGGAGC |
| *BraJAZ1* | GGCCACCTTCATAATGTCCTC | TGCTAACCCTTCCGATACCTC |
| *BraTIR1* | CACTCAACATCTCTTGCTTAGC | TTGAGAGACTTGAGATTGGGAC |
| *BraActin* | CGAAACAACTTACAACTCCA | CTCTTTGCTCATACGGTCA |
