# Supplementary material for: N-3-Oxo-Octanoyl Homoserine Lactone Primes Plant Resistance Against Necrotrophic Pathogen Pectobacterium carotovorum by Coordinating Jasmonic Acid and Auxin-Signaling Pathways
Source: Front Plant Sci. 2022 Jun 14;13:886268. doi: 10.3389/fpls.2022.886268 (PMC9237615; doi:10.3389/fpls.2022.886268)
Supplement: Supplementary file 5 [file Table_2.DOCX]

| **Supplementary Table S2 TF family primed by both 3OC8-HSL and 3OC6-HSL** [**microarray**](https://cn.bing.com/search?q=microarray&FORM=AWRE) | | | | | |
| --- | --- | --- | --- | --- | --- |
| TF family  Arabidopsis locus | 3OC6-HSL (ratio) | | 3OC8-HSL  (ratio) | Description^a^ | |
| MYB |  | |  |  | |
| At5g17300 | 57.5992 | | 26.7018 | RVE1, response to Auxin | |
| At1g19510 | 4.5109 | | 2.5629 | myb family transcription factor | |
| At3g13040 | 2.4535 | | 2.7968 | myb family transcription factor | |
| At3g50060 | 2.4358 | | 3.0545 | MYB77, response to ABA, JA, GA, SA, salt stress | |
| At5g67310 | 2.1847 | | 3.5683 | MYB44, response to ABA, JA, GA, SA, salt stress | |
| At2g23290 | 2.1564 | | 2.7372 | MYB70, response to salt stress, cadmium ion | |
| At4g00540 | 0.4827 | | 0.5 | PC-MYB2, transcription factor | |
| At1g08810 | 0.448 | | 0.4742 | MYB60, response to ABA, JA, GA, SA, salt stress | |
| At5g07690 | 0.1014 | | 0.1554 | MYB29, response to JA, GA, SA | |
| AP2 domain |  | |  |  | |
| At3g16770 | 4.6551 | | 3.4163 | ATEBP/RAP2.3, DNA binding, transcription factor | |
| At5g51190 | 4.2114 | | 3.434 | Response to chitin, ethylene-mediate signaling pathway | |
| At1g21910 | 3.1751 | | 3.8529 | Ethylene-mediate signaling pathway | |
| At2g44940 | 2.8276 | | 2.6976 | Ethylene-mediate signaling pathway | |
| At1g22190 | 2.3998 | | 2.1013 | Ethylene-mediate signaling pathway | |
| At3g58490 | 2.2457 | | 2.8238 | Phosphatidic acid phosphatase family protein | |
| At4g28140 | 0.1842 | | 0.1017 | Response to chitin, ethylene-mediate signaling pathway | |
| At5g61590 | 0.1524 | | 0.1304 | Response to water deprivation, ethylene-mediate signaling pathway | |
| NAC domain |  | |  |  | |
| At5g13180 | 3.0392 | | 3.691 | ANAC083, development | |
| At1g77450 | 2.8776 | | 3.2831 | ANAC032, development | |
| At1g01720 | 2.7307 | | 2.6101 | ATAF1, ABA-mediated signaling | |
| At3g15510 | 2.6446 | | 2.5287 | ATNAC2, development | |
| At1g69490 | 2.0457 | | 2.1308 | NAP (NAC-LIKE, ACTIVATED BY AP3/PI) | |
| At3g29035 | 0.4374 | | 0.4875 | ANAC059 | |
| At2g02450 | 0.4068 | | 0.3768 | ANAC034/ANAC035 | |
| At3g04070 | 0.3444 | | 0.108 | ANAC047, development | |
| TCP |  | |  |  | |
| At5g49480 | 5.3025 | | 3.9772 | ATCP1, calcium ion binding | |
| At2g45680 | 4.3974 | | 3.9919 | TCP family transcription factor, putative | |
| At1g55270 | 2.4632 | | 2.066 | TCP family transcription factor, putative | |
| At5g23280 | 2.3851 | | 2.4255 | TCP family transcription factor, putative | |
| At4g18390 | 0.4389 | | 0.4802 | TCP family transcription factor, putative | |
| At1g53230 | 0.3969 | | 0.3242 | TCP3, transcription factor | |
| At1g30210 | 0.3506 | | 0.3977 | TCP24; transcription factor | |
| CRF |  | |  |  | |
| At2g46310 | 4.2422 | | 5.8924 | CRF5 (CYTOKININ RESPONSE FACTOR 5) | |
| At4g23750 | 2.0276 | | 3.139 | CRF2 (CYTOKININ RESPONSE FACTOR 2) | |
| CDF |  | |  |  | |
| At3g47500 | 5.1732 | | 3.6557 | CDF3, regulation of timing of transition from vegetative to reproductive phase | |
| At5g39660 | 4.9369 | | 5.625 | CDF2, regulation of timing of transition from vegetative to reproductive phase | |
| WRKY |  | |  |  | |
| At1g68150 | | 0.4179 | | 0.3666 | WRKY9, transcription factor |
| At4g23550 | | 0.3738 | | 0.395 | WRKY29, defense response, response to chitin |
| At2g03340 | | 0.2339 | | 0.2671 | WRKY3, transcription factor |
| IAA | |  | |  |  |
| At3g23050 | | 2.5894 | | 2.6256 | IAA7, auxin mediated signal pathway |
| At4g14550 | | 2.4186 | | 2.6703 | IAA14, auxin mediated signal pathway |
| At5g54680 | | 2.2067 | | 2.0997 | ILR3, auxin mediated signal pathway |
| At4g32280 | | 0.182 | | 0.2065 | IAA29, auxin mediated signal pathway |
| Zinc finger | |  | |  |  |
| At1g26790 | | 65.9442 | | 9.3528 | Dof-type zinc finger domain-containing protein |
| At3g21150 | | 26.2804 | | 34.7247 | zinc finger (B-box type), response to chitin |
| At1g73870 | | 24.8153 | | 18.8727 | zinc finger (B-box type), regulation of transcription |
| At3g21890 | | 21.1568 | | 6.5155 | zinc finger (B-box type), response to UV-B |
| At1g24580 | | 12.3588 | | 3.5553 | zinc finger (C3HC4-type RING finger) family protein |
| At5g47610 | | 11.3691 | | 11.3019 | zinc finger (C3HC4-type RING finger) family protein |
| At4g27310 | | 11.2374 | | 10.4543 | zinc finger (B-box type) family protein |
| At4g26150 | | 10.8385 | | 11.224 | zinc finger (GATA type) family protein |
| At2g21320 | | 9.2791 | | 7.6112 | zinc finger (B-box type) family protein |
| At1g69570 | | 4.7427 | | 3.9158 | Dof-type zinc finger domain-containing protein |
| At3g55980 | | 4.2496 | | 2.8481 | zinc finger (CCCH-type) family protein |
| At5g66270 | | 4.1999 | | 5.1633 | zinc finger (CCCH-type) family protein |
| At1g20823 | | 4.1344 | | 2.783 | zinc finger (C3HC4-type RING finger) family protein |
| At1g27730 | | 3.9905 | | 2.4946 | STZ (SALT TOLERANCE ZINC FINGER) |
| At3g11110 | | 3.8138 | | 5.2425 | zinc finger (C3HC4-type RING finger) family protein |
| At3g60080 | | 3.7286 | | 3.563 | zinc finger (C3HC4-type RING finger) family protein |
| At3g52800 | | 3.544 | | 2.8718 | zinc finger (AN1-like) family protein |
| At1g49200 | | 3.4139 | | 3.6644 | zinc finger (C3HC4-type RING finger) family protein |
| At5g01880 | | 3.2795 | | 5.0778 | zinc finger (C3HC4-type RING finger) family protein |
| At4g17245 | | 3.1672 | | 4.19 | zinc finger (C3HC4-type RING finger) family protein |
| At3g10910 | | 3.1395 | | 2.6598 | zinc finger (C3HC4-type RING finger) family protein |
| At1g12440 | | 2.9618 | | 3.5478 | zinc finger (AN1-like) family protein |
| At1g51700 | | 2.9142 | | 2.4519 | ADOF1, response to chitin |
| At1g67030 | | 2.8554 | | 2.2482 | ZFP6, transcription factor |
| At5g17600 | | 2.6715 | | 3.3007 | zinc finger (C3HC4-type RING finger) family protein |
| At5g42940 | | 2.4233 | | 2.4139 | zinc finger (C3HC4-type RING finger) family protein |
| At5g27420 | | 2.3644 | | 2.7765 | zinc finger (C3HC4-type RING finger) family protein |
| At2g41940 | | 2.2474 | | 2.6676 | ZFP8, transcription factor |
| At1g25440 | | 0.4894 | | 0.3469 | zinc finger (B-box type) family protein |
| At1g32360 | | 0.4892 | | 0.4969 | zinc finger (CCCH-type) family protein |
| At5g42200 | | 0.4083 | | 0.2815 | zinc finger (C3HC4-type RING finger) family protein |
| At1g24625 | | 0.3943 | | 0.3203 | ZFP7, transcription factor |
| At1g78600 | | 0.371 | | 0.4098 | zinc finger (B-box type) family protein |
| At5g27280 | | 0.3685 | | 0.4228 | zinc finger (DNL type) family protein |
| At2g47890 | | 0.3519 | | 0.4742 | zinc finger (B-box type) family protein |
| At4g24015 | | 0.3512 | | 0.3757 | zinc finger (RING-H2 type) protein-related |
| At1g26800 | | 0.3416 | | 0.4931 | zinc finger (C3HC4-type RING finger) family protein |
| At1g68520 | | 0.3162 | | 0.338 | zinc finger (B-box type) family protein |
| At2g47890 | | 0.2857 | | 0.3367 | zinc finger (B-box type) family protein |
| At2g47890 | | 0.2624 | | 0.2371 | zinc finger (B-box type) family protein |
| At1g04990 | | 0.2242 | | 0.2931 | zinc finger (CCCH-type) family protein |
| At1g02610 | | 0.2224 | | 0.4301 | zinc finger (C3HC4-type RING finger) family protein |
| At5g63780 | | 0.2032 | | 0.1092 | zinc finger (C3HC4-type RING finger) family protein |
| At1g68190 | | 0.1704 | | 0.2839 | zinc finger (B-box type) family protein |
| At5g50450 | | 0.1349 | | 0.1788 | zinc finger (MYND type) family protein |
| At1g28050 | | 0.1178 | | 0.1473 | zinc finger (B-box type) family protein |
| At1g49230 | | 0.1158 | | 0.0853 | zinc finger (C3HC4-type RING finger) family protein |
| At5g48250 | | 0.0629 | | 0.0714 | zinc finger (B-box type) family protein |
