# Supplementary material for: N-3-Oxo-Octanoyl Homoserine Lactone Primes Plant Resistance Against Necrotrophic Pathogen Pectobacterium carotovorum by Coordinating Jasmonic Acid and Auxin-Signaling Pathways
Source: Front Plant Sci. 2022 Jun 14;13:886268. doi: 10.3389/fpls.2022.886268 (PMC9237615; doi:10.3389/fpls.2022.886268)
Supplement: Supplementary file 6 [file Table_3.DOCX]

**Supplementary Table S3. Auxin related DEGs both in 3OC8-HSL and 3OC6-HSL**

| Auxin related DEGs | 3OC6-HSL (ratio) | 3OC8-HSL (ratio) | Descriptiona |
| --- | --- | --- | --- |
| At3g12830 | 3.5821 | 3.5602 | auxin-responsive family protein |
| At1g70940 | 3.3108 | 3.7259 | PIN3 (PIN-FORMED 3); auxin hydrogen transporter |
| At4g12980 | 3.1796 | 2.3339 | auxin-responsive protein, putative |
| At3g61750 | 3.0746 | 2.6221 | auxin-responsive protein -related |
| At4g38850 | 2.6374 | 2.824 | SAUR |
| At1g72430 | 2.6197 | 3.3926 | auxin-responsive protein-related |
| At3g23050 | 2.5894 | 2.6256 | IAA7 (AUXIN RESISTANT 2); transcription factor |
| At2g38120 | 2.2419 | 2.1657 | AUX1 (AUXIN RESISTANT 1); amino acid transporter |
| At4g12550 | 2.2102 | 2.1129 | AIR1 (Auxin-Induced in Root cultures 1); lipid binding |
| At1g76270 | 0.4917 | 0.4801 | similar to putative auxin-independent growth promoter |
| At1g72420 | 0.3756 | 0.2282 | similar to auxin-induced-related |
| At2g33830 | 0.1328 | 0.1392 | dormancy/auxin associated family protein |
| At2g33830 | 0.1251 | 0.1547 | dormancy/auxin associated family protein |
